# The Composition and Effects of Chloral Hydrate

**Published:** 1883-06

**Authors:** 


### EDITORIAL, ETC.

---

THE COMPOSITION AND EFFECTS OF CHLORAL HYDRATE. —Chloral was first prepared in 1832, when Liebig, the famous chemist, obtained it by acting with chlorine gas on absolute alcohol. Its name, which consists of the first syllables of the words chlorine and alcohol, is a convenient reminder of its composition. It is a heavy, oily, colorless liquid of a specific gravity considerably greater than that of water, but boiling at a somewhat lower temperature. Among its properties may be cited a greasy, bitter taste, and a pungent odor which has an irritating effect on the eyes. Ot more interest than chloral itself is chloral hydrate, a solid crystalline substance formed by the union of chloral with a certain proportion of water. This, when dissolved in water in varying proportions, constitutes a drug extensively used, and, as is well known to the student of medical literature, not unfrequently with disastrous results. For a long time, however, chloral hydrate was "a scientific curiosity" belonging to a series of compounds much studied by Stadeler and the distinguished French chemist, M. Dumas, who showed that in a presence of an alkali, such as soda or potash, chloral hydrate is broken up into formic acid and chloroform. This was the turning point of its career, as from the fact that it could be thus decomposed in a test-tube. Another chemist, Liebreich, was led to conjecture that a similar decomposition might be produced in the blood. Experiment confirmed his anticipation, chloroform being set free in the blood and producing effects analogous to those produced when the vapor of that drug is inhaled. He accordingly, in 1869, introduced the aqueous solution of chloral hydrate to the medical profession as an anæsthetic and hypnotic. Judiciously used, under the guidance of competent physicians, it has been found to be a valuable addition to the pharmacopœia, but used to excess—as it certainly was during a brief period of great popularity—it produces a number of bad effects, to which in the aggregate the term chloralisms is applied. Chloral hydrate is of special value as a soporific when opium is inadmissible, as in the case of children and in some fevers. In cases of tetanus it is valuable as a means of securing muscular relazation, and Liebreich has shown that it produces effects in antagonism to those of strychnia. Cautiously used, it has been found serviceable in delirium tremens, rabies, acute mania, cholera, seasickness, cancer, chronic rheumatism and other diseases. The first effect of a dose of chloral hydrate is to congest the brain. Its subsequent effects vary with the individual, but, as a rule, a dose of 20 grains acts upon a healthy person as a mild sedative of the sensory nervous system, causing thirty or forty minutes after it has been taken a light normal sleep, followed by no ill consequences. Taken in large quantities, it is a powerful soporific. The temperature of the body is lowered, the force of the heart's action is diminished, and respiration is made slower. Insensibility ensues, with pallor, coldness of the extremities, lividity, muscular relaxation, and probably in the end death from cardiac syncope. Sometimes chloral hydrate fails to relieve suffering, and when it does not induce sleep may occasion excitement and delirium. To increasc the dose under such circumstances is dangerous, especially to patients affected with disease of the heart. A most important circumstance for users of the drug to take into consideration is the fact that "the limit of the dose that can be safely taken is not affected by the customary use of the drug, as in the case of opium, but rather the reverse." The habitual use of it to gain relief from sleeplessness and neuralgia is to be deprecated, as, among other bad effects, it tends to "bring on deep melancholy, weakness of will, and inability to sleep without the drug." Like all other drugs taken to gain unconsciousness of the ills of life, it is apt to be used to excess, and to prevent the acquisition of those habits of self-restraint which best conduce to sound physical health.

---

